# The influence of species identity and geographic locations on gut microbiota of small rodents

**DOI:** 10.3389/fmicb.2022.983660

**Published:** 2022-12-01

**Authors:** Zhenyu Wang, Chao Zhang, Guoliang Li, Xianfeng Yi

**Affiliations:** ^1^Nanchang Key Laboratory of Microbial Resources Exploitation & Utilization From Poyang Lake Wetland, College of Life Sciences, Jiangxi Normal University, Nanchang, China; ^2^State Key Laboratory of Integrated Management of Pest Insects and Rodents, Institute of Zoology, Chinese Academy of Sciences, Beijing, China; ^3^College of Life Sciences, Qufu Normal University, Qufu, China

**Keywords:** species identity, geographic locations, gut microbiota, rodents, Northeast China

## Abstract

Although the correlation between gut microbiota, species identity and geographic locations has long attracted the interest of scientists, to what extent species identity and geographic locations influence the gut microbiota assemblages in granivorous rodents needs further investigation. In this study, we performed a survey of gut microbial communities of four rodent species (*Apodemus agrarius*, *A. peninsulae*, *Tamias sibiricus* and *Clethrionomys rufocanus*) distributed in two areas with great distance (> 600 km apart), to assess if species identity dominates over geographic locations in shaping gut microbial profiles using 16S rRNA gene sequencing. We found that gut microbiota composition varied significantly across host species and was closely correlated with host genetics. We identified strong species identity effects on gut microbial composition, with a comparatively weaker signal of geographic provenance on the intestinal microbiota. Specifically, microbiota of one species was on average more similar to that of conspecifics living in separate sites than to members of a closely related species living in the same location. Our study suggests that both host genetics and geographical variations influence gut microbial diversity of four rodent species, which merits further investigation to reveal the patterns of phylogenetic correlation of gut microbial community assembly in mammals across multiple habitats.

## Introduction

Intestinal tracts of animals harbor diverse and complex communities of microorganisms that had profound impact on a variety of fundamental functions of different clades of hosts (Hongoh, 2010; McFall-Ngai et al., 2013; Bai et al., 2021; Yu et al., 2021). Gut microbiota is believed to affect a wide spectrum of host physiological traits and thus play a critical role in nutritional processes in the intestine by complementing the digestive capabilities of the host (Bäckhed et al., 2004; Nicholson et al., 2005; Lee and Mazmanian, 2010; Li et al., 2021). In addition to their well-documented nutritional role in herbivorous mammals, gut microbiota may have played an important role in the development and function of the immune, metabolic, endocrine, nervous and brain development as well as sexual selection in most animals (Turnbaugh and Gordon, 2009; Diaz Heijtz et al., 2011; Sarkar et al., 2016; Dinan and Cryan, 2017). A growing body of evidence has shown that changes in the composition and abundance of gut microbiota in various host animals occur throughout life due to various internal and external factors, such as host genotype, diet, social network, and host ecology (Ley et al., 2008; Muegge et al., 2011; Goertz et al., 2019; Raulo et al., 2020).

Evidence has shown that geography appears to be one of the most influential factors on gut microbiota composition in mammals (Sudakaran et al., 2012; Suzuki et al., 2018; Goertz et al., 2019). Geographical location, which potentially affect the diet of the hosts, has been shown to contribute to gut microbiota variations of humans (Rehman et al., 2016; Shin et al., 2016), mice (Suzuki et al., 2018), bats (Phillips et al., 2012), iguanas (Lankau et al., 2012), sea turtles (Scheelings et al., 2020), firebugs (Sudakaran et al., 2012), as well as honey bees (Hroncova et al., 2015). For example, Rehman et al. (2016) found pronounced geographical patterns of microbiota diversity in human gut microbiome (Rehman et al., 2016). Linnenbrink et al. (2013) has shown evidence that 16% of variations in the gut microbial communities can be attributed to geographical distance in the wild house mice across western Europe (Linnenbrink et al., 2013). These studies provide insight into the remarkably consistent correlations between microbiota composition and geographical provenance (Sudakaran et al., 2012; Suzuki et al., 2018; Goertz et al., 2019). Apart from the influence of geography, microbiome composition has also been associated to host genetics, e.g., in the wild mice, great apes and bat (Ochman et al., 2010; Mario et al., 2015; Suzuki et al., 2019; Youngblut et al., 2019; Mallott and Amato, 2021). A close relationship between the gut microbiome composition and host genetics, which has been described as phylosymbiosis, has been widely found in different taxa of animal hosts (Kohl et al., 2017; Dunaj et al., 2020; Tinker and Ottesen, 2020; Tang et al., 2021), but not in others (Ingala et al., 2018; Lutz et al., 2019; Trevelline et al., 2020). Phillips et al. (2012) has observed a non-random pattern of the microbiome composition of different members of the order Chiroptera, reflecting the important role of host genetics in shaping microbiome composition of bats (Phillips et al., 2012). A previous study focusing on primates has revealed a correlation between host genetics and their fecal microbiota of humans and four species of great apes (Ochman et al., 2010).

Given that gut microbial communities provide many physiological functions to their hosts, especially in herbivorous animals, exploring the impact of host genetics and geographical provenance in shaping microbiome diversity of wild mammals is of great importance for predicting changes of gut microbial community in mammals. Although a lot of studies have been carried out to understand the mechanisms governing the maintenance and function of gut microbial communities, existing literature mainly focused on a single host species across several localities or multiple species at a local spatial scale (McFall-Ngai et al., 2013; Mario et al., 2015; Lutz et al., 2019). To date, there has been relatively limited study highlighting the combined influence of host genetics and geography on microbial communities of mammals (Gordon and Cowling, 2003; Phillips et al., 2012), especially rodent communities that distribute in areas with great distance (Weinstein et al., 2021). Therefore, we characterized natural variations in the gut microbiota of four wild rodent species (*Apodemus agrarius*, *A. peninsulae*, *Tamias sibiricus* and *Clethrionomys rufocanus*) collected from two distant areas in eastern North China to identify specific factors (species identity and geographical provenance) associated with gut microbial composition. The main goal of our study was to determine the relative contributions of species identity and geographic locations to the proximate and ultimate causes of microbial variations in the gut microbiotas among mammal hosts in the context of host ecology and evolution.

## Materials and methods

### Animal trapping and cecal sample collection

**Figure 1:**
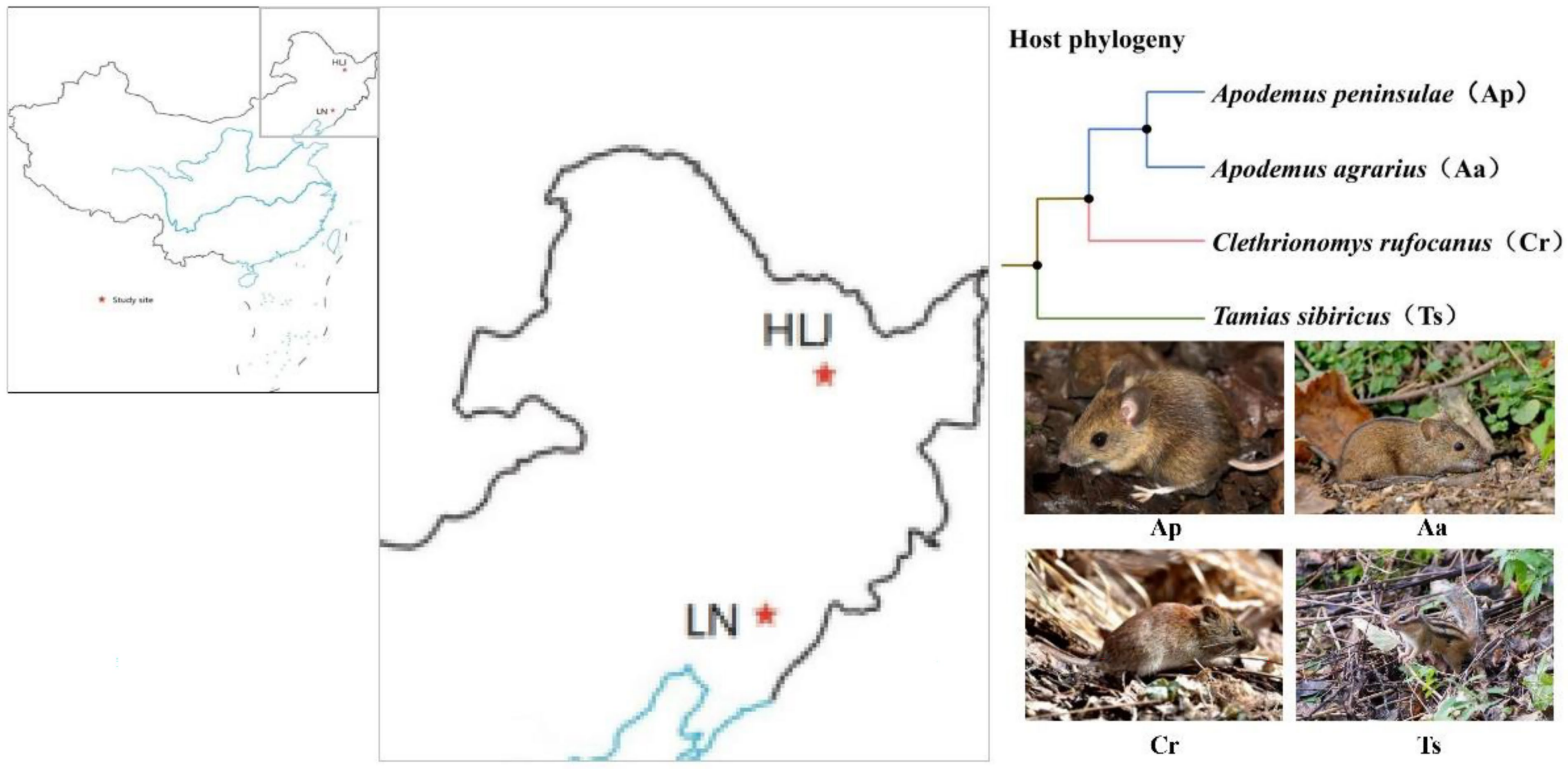
Schematic representation of the two study sites in Liaoning (LN) and Heilongjiang (HLJ) province and phylogenetic relatedness of four rodent species.

Rodents used in this study were live captured in August 2016 in two distant areas: the Qingyuan Forest Ecosystem Research Station of Chinese Ecosystem Research Network (CERN) in the eastern Liaoning Province, Northeast China (41°50 N, 124°47 E, elevation 600–800 m), and the Dongfanghong Forestry Center (46°50 N, 128°57 E, elevation 750 m) in the Dailing District, Yichun City, Heilongjiang Province, Northeast China (Figure 1). The two sites were geographically >600 km far apart from each other. The climate of the former site is a continental monsoon type with a humid and rainy summer and a cold and snowy winter. The mean annual precipitation ranges between 700 and 850 mm, 80% of which falls from June to August. Mean annual air temperature varies between 3.9 and 5.4°C with the minimum of −37.6°C in January and the maximum of 36.5°C in July. The frost-free period lasts for 130 days on average, with early frosts occurring in October and late frosts in April (Zhang and Yi, 2021). Similarly, the Dongfanghong Forestry Center is part of the north temperate zone monsoon region, with severe and long winters and short summers. The annual average temperature is 1.4°C, with a maximum of 37°C and a minimum of −40°C. Average annual precipitation is 660 mm, with 80% of annual precipitation falling between May and September (Yi and Zhang, 2008). We used live steel wire traps (9 × 10 × 25 cm; Sichuan Shujile Company, Sichuan, China) baited with peanuts to trap focal animals. The captured adult animals were immediately transferred to the lab and then scarified by cervical dislocation to collect cecal samples. In total, nine *Apodemus agrarius* (Aa_LN), seven *A. peninsulae* (Ap_LN), ten *Tamias sibiricus* (Ts_LN) and nine *Clethrionomys rufocanus* (Cr_LN) were sampled in Liaoning province. While, seven *A. agrarius* (Aa_HLJ), ten *A. peninsulae* (Ap_HLJ), eight *T. sibiricus* (Ts_HLJ) and seven *C. rufocanus* (Cr_HLJ) were collected in Heilongjiang province (Figure 1). All samples were stored at −20°C for no more than 1 week before DNA extraction.

We adhered to the ASAB/ABS Guidelines for the Use of Animals in Research for animal capture and handling in this study. The Ethical Committee of Jiangxi Normal University issued the permission to capture, handle, and maintain animals.

### DNA extraction and sequencing

Bacterial DNA from samples was extracted using the QIAamp DNA Stool Mini Kit (QIAGEN, Germany) following the instructions of manufacturer. The universal primer F (5′-GATCCTACGGGAGGCAGCA-3′) and R (5′-CCATTACCGCGGCTGCTGG-3′) was used to amplify the V3 hypervariable region of the 16S rRNA gene. The amplification reaction system (total 25 μl) contained 8.5 μl ddH_2_O, 12.5 μl Taq (2×TaKaRa), 1 μl of each primer, 2 μl DNA template. The samples were preheated at 95°C for 5 min and then amplified in a thermal cycler under the following conditions: 30 cycles of denaturation at 95°C for 30 s, annealing at 55°C for 60 s, and elongation at 72°C for 60 s, followed by a final elongation step at 72°C for 5 min. After the reaction, 2 μl PCR products were subjected to gel electrophoresis (1.5%, 100 V/60 min) to detect whether the amplification is successful. The PCR products were purified using the Agencourt AMPure XP–PCR purification kit according to the manufacturer’s instructions. Sequencing was conducted on an Illumina HiSeq PE150 platform according to protocol.

### Bioinformatic pipeline

Paired-end reads were assigned to each sample based on their unique barcode and truncated by cutting off the barcode and primer sequence. Raw sequence data were analyzed using the QIIME 2 pipeline (version 2022.2).^1^ DADA2 plugin in the QIIME2 pipeline with the denoise-paired option was used to process sequence reads. Using DADA2, raw sequence reads were quality filtered and denoised, and after which paired-end reads were merged. Merged reads were clustered into amplicon sequence variants (ASVs) after chimeric sequences removal based on sequence similarity. Taxonomy was assigned to each ASV using both the SILVA (v138) and GTDB rRNA database (v202). Taxonomy annotation from GTDB database was finally used for analyses since more ASVs were annotated. To *de novo* construct the phylogenetic tree of ASV sequences, we began by performing multiple-alignment using **DECIPHER** R package. Then FastTree was used to inferred approximately-maximum-likelihood phylogenetic tree from the alignments[x]. All data were organized and combined using **phyloseq** package for further analyses.

ASVs that were present in least than 5 samples were filtered before further analysis. To estimate alpha diversity, ASV reads were first rarefied to an equal depth to account for variability in sequencing depth. Several alpha diversity index (Observed, Shannon index, Chao1) was then estimated using *estimate_richness* function in phyloseq package.

### Statistical analysis

Non-parametric Mann–Whitney U test and Kruskal-Wallis test were performed to assess the differences in diversity indexes between two groups and among multiple groups, respectively. Read counts were converted into relative abundances and Bray–Curtis similarity matrices were generated. To assess the microbiota compositional differences between rodent species and between locations, principal coordinate analysis (PCoA) plots based on Bray–Curtis dissimilarity matrices were generated on genus relative abundances of samples for visualization. To further analyze the overall variance contribution of factor rodent species and location on gut microbiota composition, permutational analysis of variance (PERMANOVA, permutations = 999) was performed using the *adonis2* function in the **vegan** R package based on Bray-Curtis distances.

Linear discriminant analysis (LDA) effect size (LEfSe) was performed for the comparisons among rodent species group and between location groups of the sample rodent species. The LDA scores were set at a threshold >2 and used to generate histograms of enriched taxa for each group. All statistical analyses were performed using R version 4.2 unless specific illustration.

## Results

Overall, 20.95Gb raw sequence reads were obtained from 67 cecum samples of four rodent species in Liaoning and Heilongjiang province, with an average of 3,126,261 reads for each sample (ranging from 1,177,353-7,919,503). Based on rarefaction curve analysis, we found ACE, Chao1 and observed ASVs reached highly saturation (Supplementary Figure S1), which indicated that the sequencing depth was very sufficient. We suggested that 0.1 million level 16S amplicon sequence reads were enough for wild rodent species gut microbiome analysis. After a series of quality filtering, we obtained a total of 162,452,819 clean reads from all samples, averaging 2,424,669 reads per sample. A total of 2,746 ASVs were generated from all samples after the DADA2 analysis pipeline. Of these ASVs, 2,631, 1,315 and 903 ASVs were annotated by the GTDB database at the phylum, genus and species levels, respectively. Those ASVs not annotated at phylum levels were filtered before further analyses.

**Figure 2:**
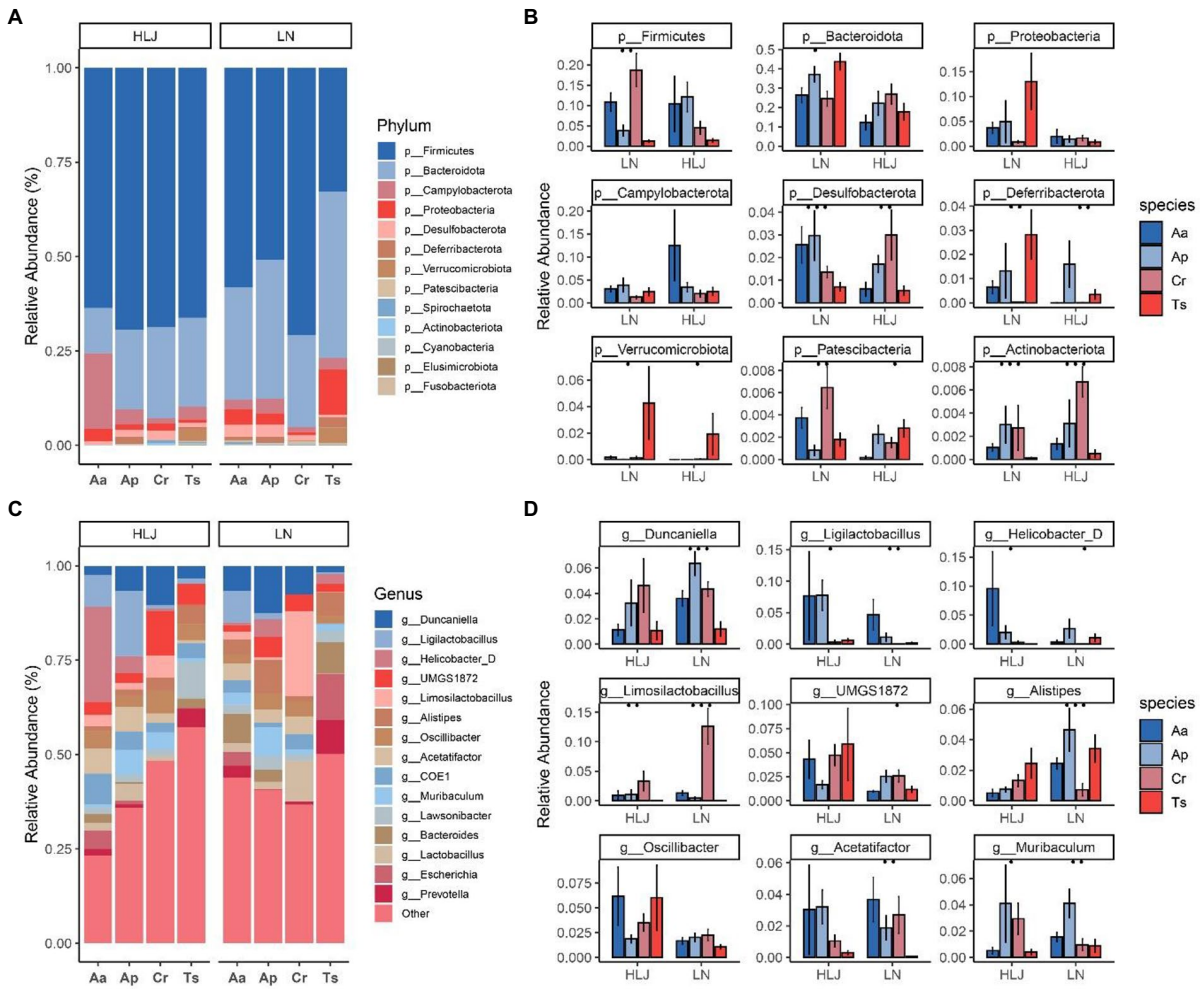
Relative abundance of bacterial phyla **(A)** and genus **(C)** in the microbiota of four rodent species. Comparison of bacterial phylum and genus of the microbiota of four rodent species that the top nine phyla **(B)** and genera **(D)** with the significant differences are shown. Aa, Ap, Ts, Cr stand for *Apodemus agrarius*, *A. peninsulae*, *Tamias sibiricus* and *Clethrionomys rufocanus*, respectively. HLJ, LN stand for Heilongjiang and Liaoning province, respectively.

At the phylum level, 98.67% of ASVs identified from the rodent cecal samples belonged to the top 10 phyla. The most abundant bacterial phylum of the four rodent species was Firmicutes (61.50 ± 21.1%), Bacteroidetes (26.8 ± 15.8%), Proteobacteria (3.75 ± 8.63%), Campilobacterota (3.65 ± 7.24%), Desulfobacterota (1.71 ± 1.89%; Figure 2A). Although microbiota composition overlapped across most host species at the phylum level, broad differences were evident. Kruskal-Wallis test analysis showed there are seven phlya significantly different among the four rodent species, including the dominant phyla Desulfobacterota (q = 0.007). Geographic locations also had some influence on the abundance of gut micobiotal at phyla level among four rodent species. In Liaoning province, that eight phyla including Firmicutes, Bacteroidetes, Desulfobacterota, Deferribacterota, Verrucomicrobiota, Patescibacteria and Actionbacteriota were significantly different among the four rodent species (all *p* < 0.01, Figure 2B). While in Heilongjiang province only five phyla (Desulfobacterota, Deferribacterota, Verrucomicrobiota, Patescibacteria and Actionbacteriota) were significantly different among the four rodent species (all *p* < 0.01, Figure 2B). The proportion of Actionbacteriota of *C. rufocanus* in Heilongjiang province was significantly higher than that in Liaoning province (HLJ vs. LN, q = 0.0445, Figure 2B). Bacteroidetes, Proteobacteria and Verrucomicrobiota in *A. agrarius* of LN group were significantly higher than that of HLJ group (HLJ vs. LN, q = 0.0367, q = 0.0404, q = 0.046, respectively, Figure 2B). Desulfobacterota in *T. sibiricus* of LN group were significantly larger than that of HLJ group (HLJ vs. LN, q = 0.0191, Figure 2B).

At the genus level, the gut microbiota of the four species were dominated by *Oscillibacter* (6.1 ± 10.15%), *Duncaniella* (5.77 ± 6.37%), *UMGS1872* (5.56 ± 7.49%), *Ligilactobacillus* (4.83 ± 11.9%), *Limosilactobacillus* (4.3 ± 8.9%), *COE1* (4.21 ± 10.52%), *Acetatifactor* (3.78 ± 6.63%), *Alistipes* (3.75 ± 4.42%), *Lawsonibacter* (3.68 ± 3.86%), *Muribaculum* (3.37 ± 5.71%; Figure 2C). Kruskal-Wallis test analysis showed there are 104 genus significantly different among the four rodent species, especially for dominant genus such as *Duncaniella*, *Ligilactobacillus*, *Limosilactobacillus*, *Acetatifactor*, *Lawsonibacter* and *Muribaculum* (q = 0.004, q = 0.0004, q < 0.001, q = 0.002876, q = 0.00770 and q = 0.0123, respectively). While geographic locations did shape the microbiota, this effect was largely within species. For instance, the proportion of *Oscillibacter* and *UMGS1872* of *A. agrarius* in HLJ was significantly higher than that in LN (q = 0.0164 and q = 0.0039, respectively; Figure 2D). *Alistipes* in *A. peninsulae* of LN group were significantly higher than that of HLJ group (q = 0.0061; Figure 2D).

**Figure 3:**
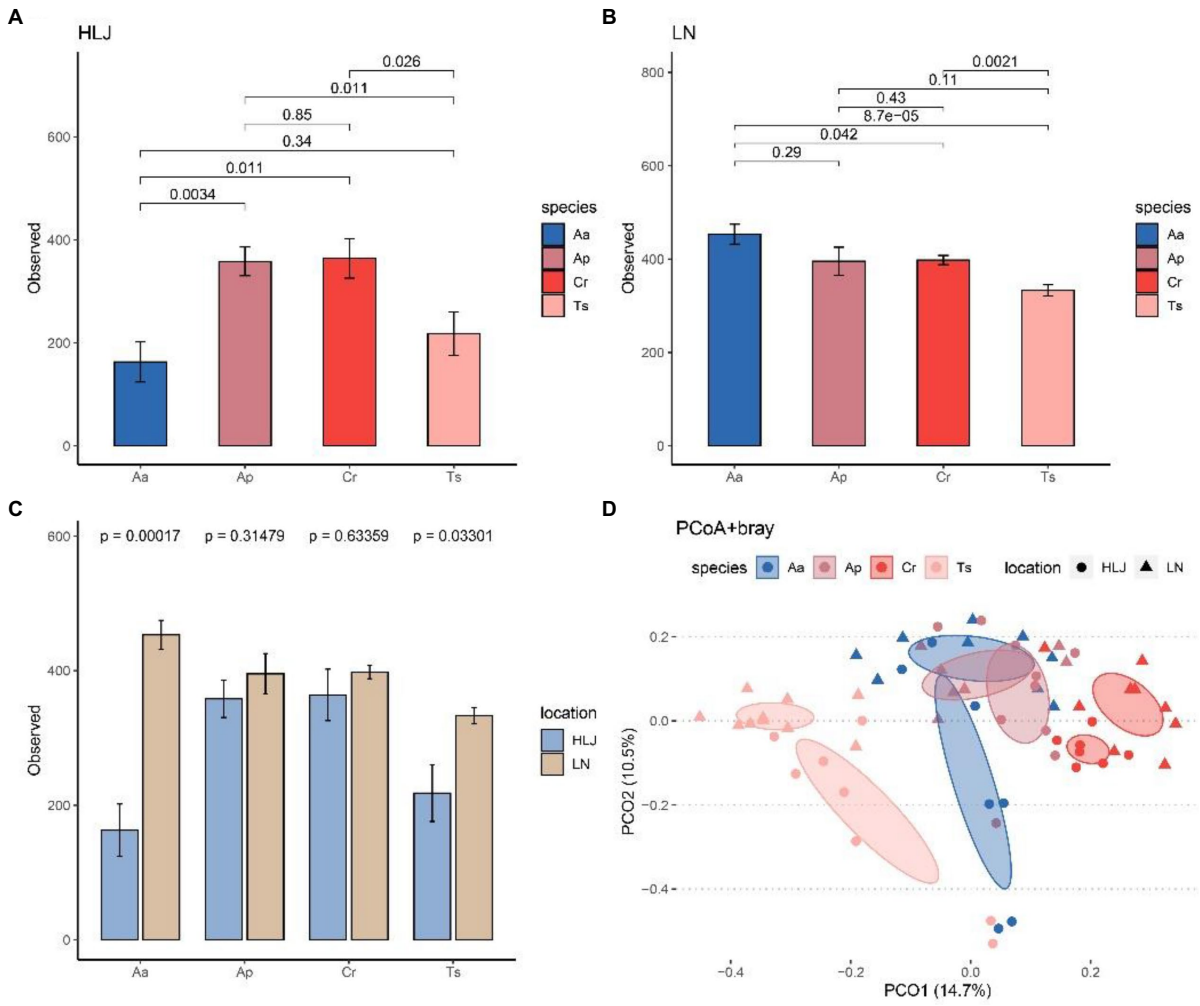
Comparison of the α-diversity (number of observed ASVs) among four rodent species in Heilongjiang **(A)** and Liaoning **(B)** province. **(C)** Comparison of number of observed ASVs for four rodent species between Heilongjiang and Liaoning province. **(D)** Principal-coordinates analysis (PCoA) based on Bray–Curtis dissimilarity matrices of the gut microbiota of four rodent species. The ellipse represents the 95% confidence level. Aa, Ap, Ts, Cr stand for *Apodemus agrarius*, *A. peninsulae*, *Tamias sibiricus* and *Clethrionomys rufocanus*, respectively. HLJ, LN stand for Heilongjiang and Liaoning province, respectively.

Alpha diversity analysis indicated that species identity had significant effect on alpha diversity (observed species) of gut microbiota at community level both in Heilongjiang (*p* < 0.001) and Liaoning province (*p* = 0.036). However, there were different patterns in these two locations. Alpha diversity of gut microbiota of *A. agrarius* in Liaoning province were significantly larger than *C. rufocanus* (*p* = 0.042) and *T. sibiricus* (*p* < 0.001; Figure 3A), while in Heilongjiang alpha diversity of *A. agrarius* were significantly smaller than *C. rufocanus* (*p* = 0.011) and *A. peninsulae* (*p* = 0.002; Figure 3B). In addition, alpha-diversity of gut microbiota of *T. sibiricus* in Liaoning province was higher than the same species in Heilongjiang (*p* = 0.021; Figure 3C).

PCoA analysis based on Bray–Curtis dissimilarity matrices at genus level showed the gut microbiota of rodents largely cluster by host species, but less so by locations. The cecal samples from the same rodent species significantly clustered together, which indicated that the gut microbial compositions among different individuals of the same species were highly similar. Furthermore, *A. peninsulae* and *A. agrarius* groups belong to the same genus of rodent were clustered more closely, thus may have more similar gut microbial composition (Figure 3D). However, the *T. sibiricus* group samples clustered separately from the other three rodent species. The cecal samples of *T. sibiricus* and *C. rufocanus* that came from two areas were separated to some extent. Results of PERMANOVA testing also showed species identity significantly explained 20.9% (*F* = 5.78, R^2^ = 0.209, *p* = 0.001) of the beta diversity between samples (as measured by Bray-Curtis distance), which was higher than that explained by geographic locations (*F* = 3.62, R^2^ = 0.044, *p* = 0.001; Figure 3D). PERMANOVA tests based on other beta diversity metrics can give similar results (Supplementary Table S1).

**Figure 4:**
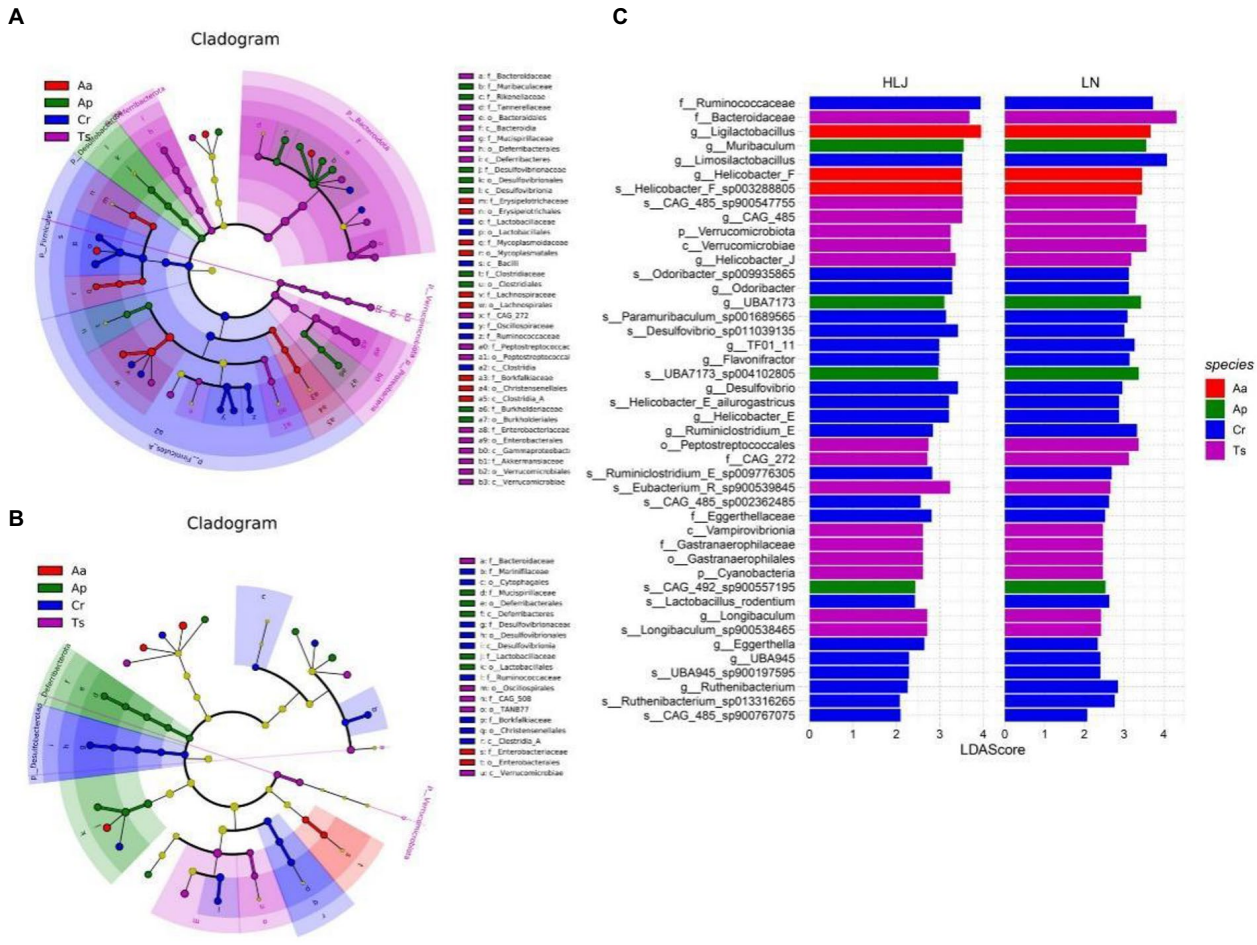
Linear discriminant analysis effect size (LEfSe) analysis. The cladogram diagram shows the microbial species with significant differences in the four species of rodents in Liaoning **(A)** and Heilongjiang **(B)** province (LDA score > 3.0). Different colors indicate different groups, with the species classification at the level of phylum, class, order, family, and genus shown from the inside to the outside. **(C)** Plot from LEfSe analysis. The plot was generated using the online LEfSe project. The length of the bar column represents the linear discriminant analysis (LDA) score. The figure shows the enriched microbial taxa that shared by conspecific rodent species between Liaoning and Heilongjiang province (LDA score > 2.0). Aa, Ap, Ts, Cr stand for *Apodemus agrarius*, *A. peninsulae*, *Tamias sibiricus* and *Clethrionomys rufocanus*, respectively, while LN and HLJ indicate rodents captured in Liaoning and Heilongjiang province, respectively.

LEfSe analysis of the gut microbial composition of the different rodent species came from Liaoning and Heilongjiang province revealed substantial differences. In Heilongjiang province, LEfSe identified nine, five, five and two taxa (LDA > 3.0) with discrepancies in relative abundance in the *C. rufocanus*, *T. sibiricus*, *A. peninsulae* and *A. agrarius*, respectively (Figure 4A). However, there were more taxa differed in relative abundance among four rodent species in Liaoning province (six, sixteen, nine and nine taxa in the *C. rufocanus*, *T. sibiricus*, *A. peninsulae* and *A. agrarius*, respectively; Figure 4B).

Based on LEfSe results, we identified representative gut microbes that were shared by conspecific rodent species in both regions (Figure 4C). Consequently, 22 taxa were shared by *C. rufocanus* came from two regions. Of them, two, ten, and ten taxa were identified at the family, genus, and species levels, respectively. For *T. sibiricus*, 14 taxa were shared, and of them, two, two, two, three, three, two taxa were identified at the phylum, class, order, family, genus, and species levels, respectively. For *A. peninsulae*, four taxa (two and two, at the genus and species levels, respectively) were shared. For *A. agrarius*, only three taxa (two and one, at the genus and species levels, respectively) were shared. Based on LEfSe results, we also identified enriched gut microbes that were shared by four rodent species in Liaoning or Heilongjiang province (Supplementary Figure S2). The results showed heterospecific rodents live in Liaoning province converged somewhat in gut microbiota composition.

## Discussion

Although it has been well accepted that gut microbes exert great influence on the ecology and evolution of mammalian hosts (Kohl et al., 2017; Ingala et al., 2018), how gut microbial composition is altered and why such variation exists in different clades of animals remains largely unknown. Here, we first reported the influence of species identity and geographical provenance on gut microbial composition of four rodent species in Northeast China, to test the relative importance of host genetics and geographical locations on the gut microbiota assemblages of sympatrically distributed mammals. We find that in four rodent species, species identity (host genetics) and geographical locations both shape gut microbiota composition. However, species identity may impose more influence on the microbiota of sympatric small mammals. Specifically, microbiota of one species was on average more similar to that of conspecifics living in separate sites than to members of a closely related species living in the same location.

We observed considerable variations in the gut microbial composition at interspecific level despite sympatric distribution of rodents in Liaoning or Heilongjiang province. These observations were consistent with the results of previous studies showing a close correlation between the similarity of their microbial composition and the phylogenetic relationships of the hosts (Phillips et al., 2012; Mario et al., 2015; Brooks et al., 2016; Rojas et al., 2021). PCoA analyses found species identity significantly explained 20.9% of the beta diversity between samples, which was much higher than that explained by geographic location. Thus, this may provide evidence that species identity showed more effects on the gut microbiota of the four rodent species, suggesting that closely related hosts harbored more similar gut microbial communities than divergent ones (Phillips et al., 2012). The fact that gut composition of the four rodent species both at phylum and genus level were affected by species identity rather than by geographical locations conveyed a strong phylogenetic signal on microbial composition of rodents. It was similar with previous research which has shown that host genetic factors dominate over geography and dietary niche in shaping the gut microbiota of primates (Amato et al., 2016), although members of species living in closer geographic proximity (Moeller et al., 2013). Since host genetics has been found to be an important factor in shaping gut microbial composition of animals (Ley et al., 2008), we also detected a significant topological congruence between the gut microbiome composition and host genetics, i.e., the similarity of gut microbial communities of rodents paralleled their phylogeny. Gut microbiota of individuals of the same genera (*A. agrarius* and *A. peninsulae*) was clustered together in regardless of geographical difference, providing further evidence for strong phylosymbiosis widely existing in different clades of animal hosts (Kohl et al., 2017; Tinker and Ottesen, 2020; Tang et al., 2021).

Phylosymbiosis conveys the idea that genetic backgrounds of the host animals are closely related to changes in their gut microbial diversity (Brooks et al., 2016). However, host genetics and host ecology may structure the gut microbiota of mammals at different taxonomic scales (Rojas et al., 2021). Diet has been believed to be a crucial factor in structuring gut microbial diversity in the context of host ecology. Previous studies have shown that the diversity of gut microbiota of some clades of animals changed significantly in response to host diet (Muegge et al., 2011; Colman et al., 2012; Weldon et al., 2015). In our study, herbivorous *C. rufocanus* with plant-based diets (e.g., seeds, roots and bark) showed a more clustered arrangement of their gut bacterial components despite with relative higher diverse microbiomes, suggesting that fiber-based diets may show less specificity in microbiome compositions. However, seed-based feeders *T. sibiricus* exhibited relatively low diversity microbiomes, possibly reflecting their seed hoarding behavior across the study areas (Yi and Zhang, 2008; Zhang and Yi, 2021). Although the detailed information of diets was not clear for the four rodents, we are unable to rule out the possibility that differences in diet composition play a role in the assembly of gut microbiomes in Rodentia. Diet could change as a result of phylogenetic effects that host species have evolved different dietary preferences or environmental effects that hosts feeding different things in different habitats depend on local food sources. Therefore, dietary variation could contribute to microbiota differences across species, capture sites, or both. However, once the effects of host genetics were excluded from the interactive analyses, geographic locations alone failed to influence the intestinal bacterial components, indicating that host genetics may be more influential than geographic locations in shaping the gut microbiota of closely related host species.

Consistent with the observations in previous studies of host-microbe associations showing that gut microbiota of house mice (*Mus musculus*) differed significantly in alpha diversity in response to geographical locations (Weldon et al., 2015; Suzuki et al., 2018; Goertz et al., 2019), We also found the role of geographical provenance in shaping gut microbial diversity of the four rodent species. For instance, alpha diversity of gut microbiota of *A. agrarius* in Liaoning province were significantly larger than *C. rufocanus* and *T. sibiricus*, while in Heilongjiang alpha diversity of *A. agrarius* were significantly smaller than *C. rufocanus* and *A. peninsulae*. Alpha-diversity of gut microbiota of *T. sibiricus* in Liaoning province was higher than the same species in Heilongjiang. Moreover, the gut microbial composition of the four rodent species did change significantly in response to geographical variation either at the phylum or genus level. While geographic locations did shape the microbiota, this effect was largely within species; heterospecific small rodents converged somewhat in gut microbiota composition when living in sympatry (especially in Liaoning province, Supplementary Figure S2), but this was insufficient to override the strong influence of species identity. The climate conditions and vegetation composition appeared to be similar although the two locations were geographically >600 km far apart each other (Yi and Zhang, 2008; Zhang and Yi, 2021). It can be expected that rodent species distributed in the two distant areas may rely on similar staple food resource (e.g., seeds and other plant parts), which may also play a role in altering gut microbial compositions of mammals (Muegge et al., 2011; Kohl et al., 2017).

Overall, our study provided the first large-scale profiling of the gut microbiome of four rodent species in two distant areas in China. Our results suggest that host genetics and geographic locations both serve as major driving forces in shaping gut microbial diversification of the wild animals at a large scale of geographic isolation. Our findings also provide avenues for better understanding of the driving forces mediating the symbiosis between microflora communities and hosts.

## Data availability statement

The data presented in the study are deposited in the NCBI repository, accession number PRJNA902417.

## Ethics statement

The animal study was reviewed and approved by the Ethical Committee of Jiangxi Normal University.

## Author contributions

ZW and XY designed the experiments and wrote the manuscript. ZW performed the experiments. ZW, CZ, GL, and XY performed the data analysis. All authors contributed to the article and approved the submitted version.

## Funding

This study was supported by the National Natural Science Foundation of China (32070447 and 32160244), the Youth Talent Introduction and Education Program of Shandong Province (20190601), the Opening Fund of Key Laboratory of Poyang Lake Wetland and Watershed Research (Jiangxi Normal University), Ministry of Education (PK2018005) and Project of Education Department of Jiangxi Province (GJJ150304).

## Conflict of interest

The authors declare that the research was conducted in the absence of any commercial or financial relationships that could be construed as a potential conflict of interest.

## Publisher’s note

All claims expressed in this article are solely those of the authors and do not necessarily represent those of their affiliated organizations, or those of the publisher, the editors and the reviewers. Any product that may be evaluated in this article, or claim that may be made by its manufacturer, is not guaranteed or endorsed by the publisher.

## Supplementary material

The Supplementary material for this article can be found online at: https://www.frontiersin.org/articles/10.3389/fmicb.2022.983660/full#supplementary-material

## Footnotes

^1^ https://qiime2.org/
