# Supplementary material for: The influence of species identity and geographic locations on gut microbiota of small rodents
Source: Front Microbiol. 2022 Dec 1;13:983660. doi: 10.3389/fmicb.2022.983660 (PMC9751661; doi:10.3389/fmicb.2022.983660)
Supplement: Supplementary file 1 [file Data_Sheet_1.PDF]

SUPPLEMENTARY MATERIAL

FIGURE S1 Rarefaction curves of bacterial popolations in the analyzed samples. Aa-LN, Aa-HLJ, Ap-LN, Ap-HLJ, Ts-LN, Ts-HLJ, Cr-LN and Cr-HLJ stand for *Apodemus agrarius*, *A. peninsulae*, *Tamias sibiricus* and *Clethrionomys rufocanus* captured in Liaoning (LN) and Heilongjiang (HLJ) province.

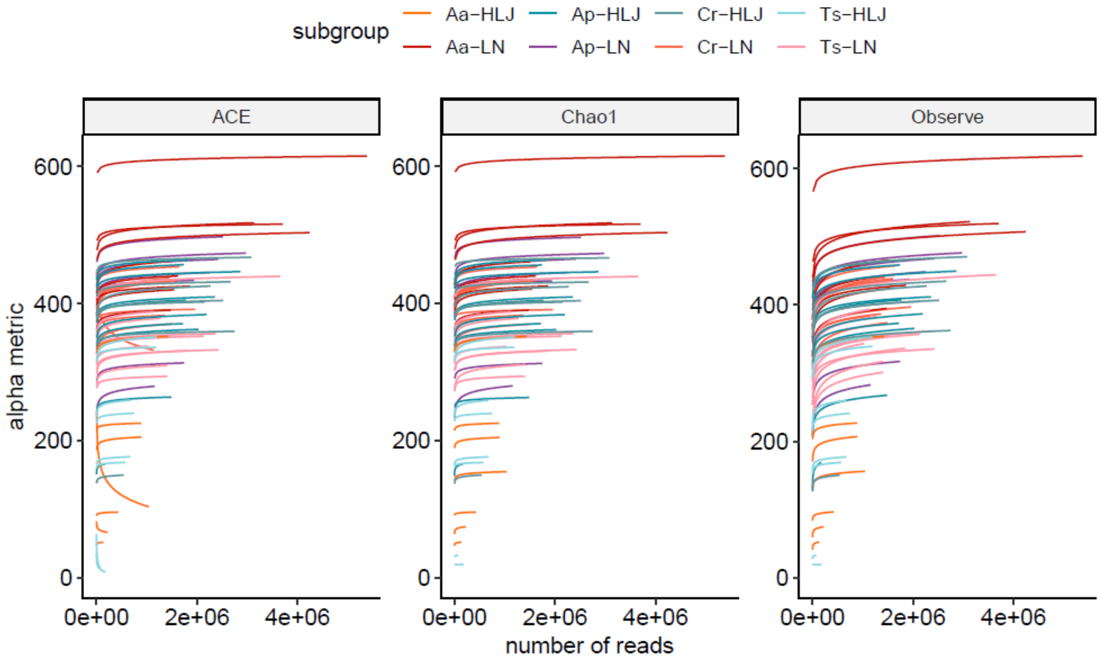

FIGURE S2 Plot from LEfSe analysis. The plot was generated using the online LEfSe project. The length of the bar column represents the linear discriminant analysis (LDA) score. The figure shows the enriched microbial taxa that shared by four rodent species in Liaoning or Heilongjiang province (LDA score > 2.0). Aa, Ap, Ts, Cr stand for *Apodemus agrarius*, *A. peninsulae*, *Tamias sibiricus* and *Clethrionomys rufocanus*, respectively, while LN and HLJ indicate rodents captured in Liaoning and Heilongjiang province, respectively.

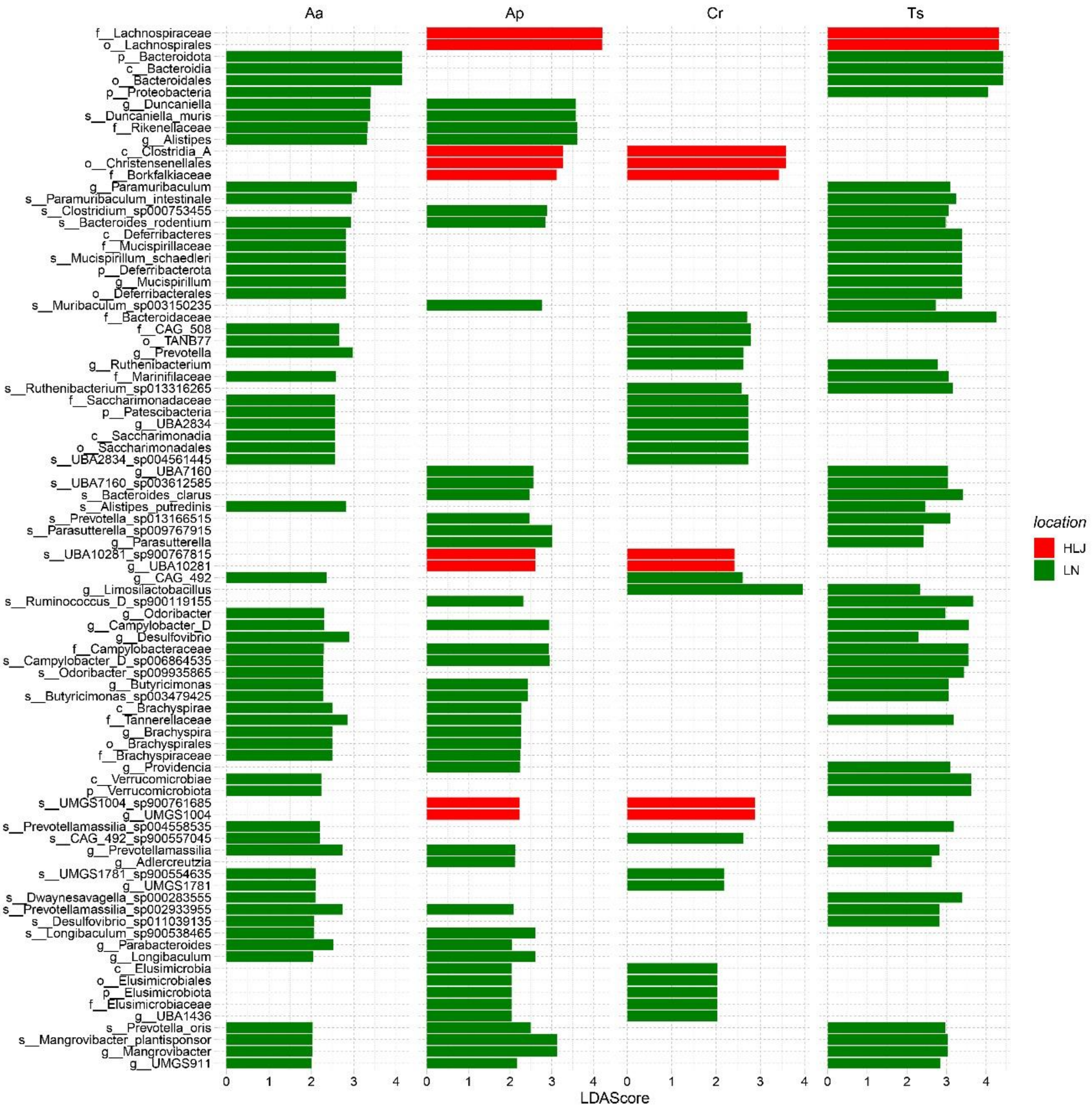

TABLE S1 Results of PERMANOVA tests examining the effects of species identity and geographic locations on three beta diversity metrics.

	Unweighted Unifrac			Weighted Unifrac			Jaccard		
	F	*P*	R^2	F	*P*	R^2	F	*P*	R^2
Geographic locations	14.3	<0.001	0.15	4.6	<0.01	0.06	2.6	<0.001	0.03
Species identity	5.7	<0.001	0.18	3.5	<0.001	0.14	3.9	<0.001	0.15
